# Is ATP a signaling regulator for postharvest chilling tolerance in fruits?

**DOI:** 10.1093/hr/uhae204

**Published:** 2024-07-26

**Authors:** Hansika Sati, Harinder Singh Oberoi, Sunil Pareek

**Affiliations:** Department of Agriculture and Environmental Sciences, National Institute of Food Technology Entrepreneurship and Management, Kundli, Sonepat, Haryana 131028 India; Department of Basic and Applied Sciences, National Institute of Food Technology Entrepreneurship and Management, Kundli, Sonepat, Haryana 131028 India; Department of Agriculture and Environmental Sciences, National Institute of Food Technology Entrepreneurship and Management, Kundli, Sonepat, Haryana 131028 India

## Abstract

Low-temperature storage is used to extend the shelf life of fruits, but prolonged storage at temperatures below tolerable levels may cause postharvest chilling injury (PCI) in sensitive commodities. This review aims to highlight adenosine triphosphate (ATP) activation and the interplay of extracellular ATP (eATP) and intracellular ATP (iATP) in fruits and to find out its significance in mitigating PCI. Various pathways, such as the Embden–Meyerhof–Parnas pathway, the tricarboxylic acid cycle, the pentose phosphate pathway, the γ-aminobutyric acid shunt pathway, and the cytochrome pathway, are studied critically to elucidate their role in continuous ATP supply and maintaining the membrane fluidity and integrity. This review summarizes the treatments helpful in modulating energy metabolism in fruit. Additionally, this work provides insights into the energy status in attenuating chilling tolerance. Moreover, it states the potential of nicotinamide adenine dinucleotide in mitigating PCI. Furthermore, it discusses the role of eATP and its receptor *DORN1* in mitigating chilling stress.

## Introduction

Fresh horticultural produce is highly perishable and susceptible to various biotic and abiotic stresses leading to postharvest diseases and disorders that affect their quality and shelf life [1]. Postharvest chilling injury (PCI) is one such stress that hampers the fruit shelf-life. Storage of horticultural produce at low-temperatures is becoming a necessity, however, continuous storage of the chilling-sensitive produce at temperatures below its critical temperature leads to the development of PCI [2]. Many chemical treatments, such as oxalic acid (OA) and salicylic acid (SA), nitric oxide (NO), chitosan, methyl jasmonate, and melatonin (MT) have been used to mitigate chilling stress [3, 4]. PCI disrupts cellular homeostasis and causes oxidative stress by disproportionately increasing reactive oxygen species (ROS) production. This leads to lipid peroxidation, DNA damage, and degradation of proteins [5]. PCI leads to decoupling reactions in mitochondria and chloroplast, which results in changes in the structure of membrane proteins involved in electron transport chain (ETC). This inhibits the mitochondrial respiration and ATPase activity, leading to a sharp decline in ATP production [6].

**Figure 1:**
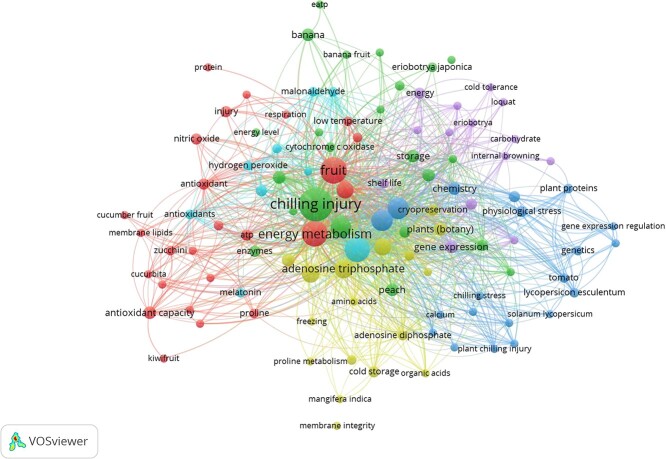
The VOS viewer software-based network visualization of keyword co-occurrence in extracted peer-reviewed scientific papers related to ATP regulation in postharvest chilling tolerance in fruits from Scopus database. The connecting lines between phrases show co-occurrences in the same article, and the keywords contained in the same cluster reveal that they have been analyzed frequently in the same publications. ATP, Adenine triphosphate

When a chilling-sensitive commodity is exposed to temperatures lower than its tolerable level for a long duration, the lipid matrix experiences increased micro-viscosity due to reduced mobility of phospholipids and unsaturated fatty acids (unSFA) [7]. This makes the gel structure rigid, resulting in reduced membrane elasticity, electrolyte leakage, membrane rupture, membrane protein dysfunction, and reduced ionic gradients [8]. In addition to the plasma membrane, other membranes are also affected by low-temperature stress; for example, it has been reported that the mitochondrial membranes undergo a tremendous phase transition in pears stored at 0± 0.5°C [9]. Similar changes were observed in tomatoes stored at 12°C [10] and cucumbers stored at 4°C [11]. These changes in the composition of the lipids in the mitochondrial membranes are responsible for influencing respiration. Changes in respiration result in increased metabolic energy loss by the affected cells [12]. This reduction in the mitochondrial oxidation rate leads to an accumulation of metabolic intermediates, leading to a reduced supply of ATP. This further results in oxidative phosphorylation decoupling, a decrease in cytochrome c oxidase (CCO) activity, and an increase in alternative oxidase (AOX) activity. This respiratory activity disintegration and metabolic dysfunction occurring in the mitochondria leads to the formation of ROS, which increases oxidative stress [13]. If the exposure time to low-temperatures is short, respiration rate will not be adversely affected and, thus, there will be no effect on oxidative phosphorylation. The production of iATP is supported by the γ-aminobutyric acid (GABA) shunt, certain respiratory pathways, the ADP/ATP carrier (AAC), mitochondrial uncoupling proteins (UCPs), and sucrose nonfermenting (SNF)-related kinase (*SnRKs*), all of which help ensure there is enough ATP [14].

Even though PCI in fruits has been extensively investigated, its exact mechanisms are still being explored. Studies have mainly reported the enzymatic and non-enzymatic antioxidant systems, proline involvement, GABA pathways, and polyamines involvement [15]. Last decade has witnessed publications reporting beneficial effects and positive commercial use of ATP to preserve fruits. Nevertheless, owing to the diversity of crop sciences, cold-storage conditions, and optimum storage conditions for different fruits and treatments being used to extend the shelf life of the fruits, a comprehensive investigation is needed to analyze and evaluate the significance of ATP in alleviating chilling stress. Therefore, this review focuses on identifying whether ATP proves to be a savior for the horticultural commodity prone to PCI. It discusses the mechanism of PCI, the effect of energy status in chilling injury and postharvest shelf-life of fruits, and explores the effect of various treatments, such as methyl jasmonate DL-β-aminobutyric acid (BABA), 1-methylcyclopropene (1-MCP), glycine betaine, brassinosteroids, 6-benzyl amino purine (6-BA), hydrogen sulfide, fibroin, trehalose, OA, NO, and MT in mitigating chilling stress by affecting the energy status.

## Methodology

In order to collect the available literature on ATP as a signaling molecule in mitigating postharvest PCI, a total of 48 peer reviewed scientific papers, including 44 articles, 2 review papers, and 2 conference papers were identified from the Scopus database by using keywords ‘ATP’ AND ‘Chilling injury’, and ‘Fruits’. All documents were limited to 2 subject areas, first being ‘Agriculture and Biological Sciences’ and the other being ‘Biochemistry, Genetics, and Molecular Biology’. The data ranges between year 2015 and 2024. A software called ‘VOSviewer’ was used for performing bibliometric analysis. It was used to develop a network analysis (Fig. 1) by mapping all keywords with minimum occurrence of 2. A total of 108 keywords (items) were identified with 6 clusters, each represented in a different color. The map represents items in the form of circles, where the size of the circle determines the weight of the item. The total links identified were 2021. The links in the figure represent the connection between the items. The clusters represent a set of closely related keywords.

Cluster 1 in red denotes that treatments like H_2_S and NO_2_ have successfully mitigated chilling stress by maintaining sufficient ATP supply. These studies have mainly focused on lowering malondialdehyde (MDA) levels and membrane degradation and enhancing the antioxidant capacity of low-temperature affected fruits. Cluster 2 in green represents studies on ATP levels conducted on fruits stored at low-temperatures at the genomic levels. Cluster 3 in navy blue shows research focused on cold-stored tomatoes, for evaluating the ATP levels. Cluster 4 in golden yellow shows ATP-related studies conducted on cold-stored mangoes. Cluster 5 in purple shows lower ATP levels in loquat, which caused CI signs like internal browning. Lastly, cluster 6 in light blue denotes studies focusing on ATP in chilling-affected fruits to be associated with elevated ROS generation. The clusters 1, 2, 3, 4, 5, and 6 comprise 25, 23, 20, 17, 14, and 9 items, respectively. As 6 clusters are available for the stated topic, this review attempts to summarize the scattered literature and provide critical insights.

### Plant respiratory pathways strengthen chilling tolerance

**Figure 2:**
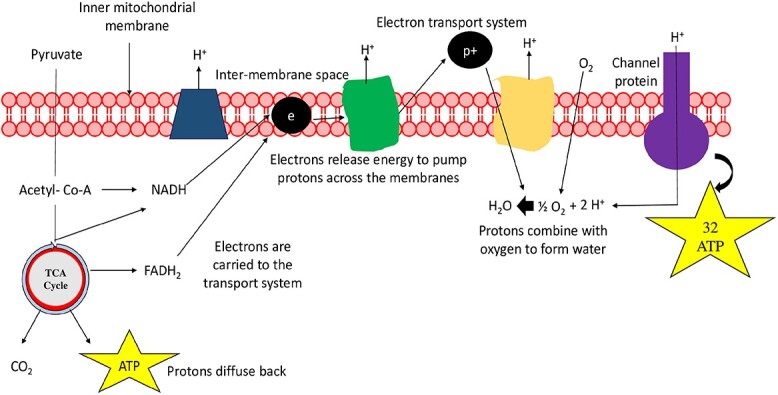
An overview of ATP synthesis in the mitochondrial membrane where FADH_2_, ATP, and CO_2_ are produced in the TCA cycle. Further, the electrons are harvested from NADH and FADH_2_ and carried via the TCA cycle to the transport system, providing energy to pump electrons across the membrane. ATP, Adenine triphosphate

Studies postulate that respiration in plants is associated with energy maintenance in postharvest produce. The plant respiratory pathways play an important part in the context of PCI and ATP generation [16]. Embden–Meyerhof–Parnas (EMP), GABA shunt pathway, arginine pathway, tricarboxylic acid cycle (TCA), cytochrome pathway, and pentose phosphate pathway (PPP) are the respiratory pathways in plants that play a pivotal role in ATP generation in plants. The PCI hampers the plant’s metabolic functioning and leads to alterations in plant functioning [17]. Hence, these respiratory pathways are paramount when fruits encounter chilling stress. They help meet the increasing demand by generating ATP, enabling plants to withstand chilling stress (Fig. 2).

### Embden–Meyerhof–Parnas pathway

Embden–Meyerhof–Parnas, commonly known as the EMP pathway or glycolysis, refers to the formation of pyruvate from glucose. A number of kinases are responsible for catalyzing the various reactions of this pathway. The first reaction is catalyzed by the enzyme hexokinase, which converts glucose to glucose-6-phosphate, an isomer of fructose-6-phosphate. The second step is catalyzed by phospho-fructo-kinase (PFK), one key enzyme in the pathway, which forms fructose-1-6-diphosphate. This enzyme is responsible for energy control in plant cells, since excess ATP leads to its inhibition. This is followed by a series of reactions leading to the formation of pyruvate by breaking six-carbon glucose into three-carbon glyceraldehyde. EMP produces energy associated with the maintenance of cell membrane integrity [18]. OA-treated peach has been documented to show an enhanced EMP pathway that provides energy to maintain cellular integrity, contributing to chilling tolerance [18]. However, limited studies have reported that PCI in fruits is a complex and multifaceted phenomenon; thus, exploring the role of EMP pathway in PCI-affected fruits demands disentangling its interactions with other metabolic pathways and stress-responsive mechanisms, which might be challenging and resource-intensive.

### Tricarboxylic acid cycle

Pyruvate generated in the EMP pathway is broken down into water and CO_2_ through a series of complex reactions in the TCA cycle, also known as Krebs cycle, resulting in the formation of ATP molecules. These steps involve several enzymes and coenzymes. The action of dehydrogenase enzymes traps most of the energy as they assist in the removal of a hydrogen molecule and the addition of water. This complex process occurs through the transfer of electrons in the mitochondria through ETC. The combined processes of glycolysis and the TCA cycle led to a net gain of approximately 32 ATPs [19]. Guanosine triphosphate (GTP), nicotinamide adenine dinucleotide (NAD), guanosine diphosphate (GDP), nicotinamide adenine dinucleotide phosphate (NADP), flavin adenine dinucleotide (FAD) are specific nucleotides responsible for the above transitions [20].

The conversion of NAD to FAD leads to the generation of 3 ATPs, from the step of FAD, 2 ATPs are generated. Besides providing energy, the TCA cycle is also responsible for generating fumaric, citric, oxalic, and succinic acids in the form of the carbon skeleton as in the case of calcium chloride-treated cold-stored pineapples [20]. These organic acids and carbohydrates are utilized instead of proteins and fats and form the basic substrates used by plant tissues for respiration. However, these complex macromolecules can be broken down into simpler substances in the TCA cycle. Studies conducted by Tsuchida et al. [21] on cucumbers cv. ‘Toppugurin’ stored at 1°C experienced increased incorporation of acetate-1,2-^14^C into the TCA cycle as a measure of pyruvate utilization, followed by which the cold-stored cucumbers were then reported to have lower citrate synthase and higher alanine and pyruvic acid activity. Thus, these enzymes were reported to be the biomarkers of stress-induced tolerance.

MT treatment of 400 μM to litchi stored at 5°C experienced higher chilling tolerance than control. By keeping the fruit at an average temperature after the end of refrigeration, the enzymatic activity of CCO, succinate dehydrogenase (SDH), H^+^-ATPase, and Ca^2 + -^ATPase declined in both groups. However, it declined less in MT-treated litchi than in the control. This lower decline in the activity of energy-related enzymes of TCA cycle was the cause of higher energy in the MT-treated litchi [22]. Another study on bell peppers stored at 1°C revealed that the appearance of PCI symptoms was attributed to the decline in proteins associated with TCA cycle [23]. Hydrogen sulfide has also been studied to mitigate PCI in peaches stored at 5°C by regulating the TCA cycle. The EMP pathway oxidizes glucose to pyruvate followed by TCA cycle converting it into water and ATP [24]. A study by Sun et al. [25] on Chinese olive stored at 2°C documented PCI symptoms as PCI affected the normal functioning of TCA cycle and led to lower ATP generation.

### Pentose phosphate pathway

Another important pathway for glucose metabolism is the PPP. A hexose (glucose) enters the PPP cycle and exists in the form of a pentose. As many times as the cycle is repeated, only a single carbon atom is removed to form CO_2_ or, in other words, out of six glucose molecules, only a single molecule forms CO_2_ and H_2_O [26]. Several intermediate products, such as erythrose 4-phosphate and ribose 5-phosphate, are provided by the PPP to carry out aromatic amino acid synthesis [27]. To carry out nucleotide synthesis, the PPP supplies 5-carbon skeletons (pentoses), which is considered to be an important physiological pathway. Studies have shown that peaches treated with NO showed improved tolerance to PCI by activating the PPP [28].

Pan et al. (2017) conducted an experiment to observe the effect of PCI on papaya stored at temperatures of 1°C and 6°C, and found that papaya stored at 1°C experienced more PCI signs than the fruit stored at 6°C [29]. Following this, [30] studied the effect of PPP on the chilling tolerance of papaya stored at 1°C. They noted an enhanced respiration rate as an increased PPP contributed to an increased energy supply by accumulating NADPH, thus increasing ATP production to combat PCI and provided tolerance to chilling stress [31]. Exogenous MT administered to cold-stored pomegranates has resulted in increased G6PDH and 6-phosphogluconate dehydrogenase (6PGDH) activity, which contributes to adequate intracellular NADPH accumulation [32]. Application of phytosulfokine α (PSKα) to strawberry has been shown to enhance the PPP, thereby providing NADPH, which releases intracellular ATP to combat the chilling stress [33].

### The γ-aminobutyric acid shunt pathway

Gama aminobutyric acid (GABA) ABA, a four-carbon non-protein amino acid, has been shown to play an important role against biotic and abiotic stress [34]. Polyamine oxidase (PAO) and diamine oxidase (DAO) are key enzymes known for the accumulation of GABA and polyamines, which have been reported to have alleviating effects on PCI [35]. The GABA shunt pathway involves three key enzymes, namely succinic semi-aldehyde dehydrogenase (SSADH), glutamate decarboxylase (GAD), and GABA-transaminase (GABA-T). GABA accumulation is said to be directly proportional to GAD activity and inversely proportional to GABA-T activity. GABA-T and SSADH play an important role in the TCA and ETC pathways, since they are responsible for supplying both nicotinamide adenine dinucleotide hydrogen (NADH) and succinate. An uptick in these cycles results in a greater generation of carbon skeletons, ATP, and a reduction in the accumulation of H_2_O_2_. This helps maintain the cell membrane integrity, lowers oxidative stress, increases GABA shunt, and provides more energy and, therefore, more strength to cope with the chilling stress [36]. It has been observed that MT-treated tomatoes [37], mangoes [38], and zucchini [39], experience higher GABA shunt pathway activity, which serves as a supplier of carbon skeleton and iATP molecules, to carry out phenylpropanoid pathway activity, thereby mitigating PCI.

A 1- or 5-mM direct application of GABA has also shown a positive effect on mitigating PCI in papayas stored at 4°C for 5 weeks. The exogenous GABA enhanced the enzymatic activities of CAT, APX, SOD, GR, and PAL which act as antioxidants and preserve the fruit energy. The GABA-T converts available GABA into succinate, which in turn produces ATP via ETC. Thus, GABA shunt pathway serves to be an essential phenomenon in regulating respiratory activities and ensuring adequate ATP supply [40]. A similar result was obtained in 5 mM GABA-treated peaches stored at 1°C for 5 weeks [41].

### Arginine pathway

Arginine is a proteinogenic amino acid, which also plays a multifunctional role in the biosynthesis of bioactive molecules, such as spermine, putrescine, spermidine, NO, and proline. The three key enzymes, arginine decarboxylase (ADC), arginase, and NO synthase (NOS) help in catalyzing arginine into ornithine, which acts as a precursor to proline and various polyamines [26]. Arginine leads to the formation of GABA which plays an important role in maintaining energy levels by supplying ATP in cold-stored postharvest produce in protecting them from PCI, as is the case in MT-treated tomatoes [42]. Banana treated with NO has shown lower susceptibility to PCI than the control, which is supported by elevated levels of GABA, NO, and polyamine. These elevated levels lead to elevated respiratory rates, producing adequate ATP levels [35]. Methyl jasmonate-treated cherry tomatoes have been reported to experience an activation of the arginine pathway. GABA being a primary metabolite for arginine production helps in maintaining adequate ATP levels via ETC, thus providing energy for chilling tolerance [43]. Hawthorn fruit treated with glycine betaine stored at 1°C also experienced the same as it contributed to elevating the arginine pathway [44].

### Cytochrome pathway and alternative pathway

The cytochrome pathway and alternative pathway are part of the electron transport pathway, which are essential for ATP generation and resistance to PCI. Comprising of several series of protein complexes located in the inner plasma membrane, the cytochrome pathway transfers electrons from electron donors, namely NAD and FADH_2_ to electron acceptors, namely molecular oxygen. As electrons are passed via protein complexes of the cytochrome pathway, ATP is released. This ATP is the energy currency of fruits to tolerate chilling stress [45]. Application of NO to plants has been reported to increase cytochrome oxidase activity in mitochondria. CCO is the site for the generation of ATP molecules, which proves beneficial during chilling stress [46]. It has been studied that cold-stored peaches with OA experience reduced effects of chilling stress due to high ATP generation by the cytochrome pathway [18].

Methyl SA and methyl jasmonic acid-treated sweet peppers have contributed to initiating the cytochrome pathways that lead to ATP generation. This helps in maintaining the fruit quality by protecting it against PCI [47]. Exogenous progesterone treatment in bananas has also been observed to induce an increased cytochrome pathway activity, thereby leading to ATP generation [48]. The alternate pathway, also known as the alternate oxidase pathway, is also crucial in maintaining chilling tolerance. The NO_2_-treated peaches stored at 4°C experienced upregulated AOX genes. The upregulated AOX genes relax the highly coupled and tensed ETC and maintain metabolic functioning by generating appropriate ATPs and reducing oxygen to water, thus lowering PCI [28]. However, further studies are required on alternate pathways to elucidate the operation of the AOX pathway in chilling-affected tissues of fruits. Omics research can provide new insights into unraveling the underlying molecular mechanisms involved in sufficient ATP generation via an alternate pathway in mitigating PCI.

### Enhancing cold tolerance through various treatments by modulating energy metabolism in fruits

**Figure 3:**
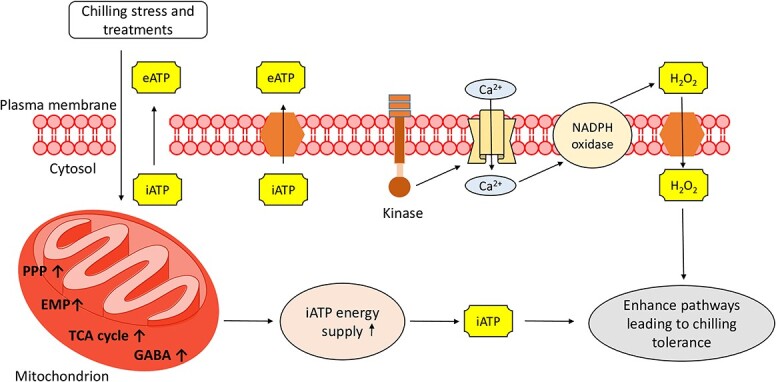
Influence of different postharvest treatments and eATP on the cold-stored produce to mitigate postharvest chilling injury. Various treatments, like fibroin, MT, NO_2,_ H_2_S, methyl jasmonate, BABA, 1-MCP, glycine betaine, brassinosteroids, 6-BA, trehalose, and OA aid in enhancing GABA shunt pathway, TCA cycle, EMP pathway, and PPP which help in maintain sufficient iATP supply to provide chilling tolerance. Abbreviations: eATP, external adenosine triphosphate; EMP, Embden–Meyerhof–Parnas pathway; GABA, γ-aminobutyric acid; iATP, internal adenosine triphosphate; PPP, pentose phosphate pathway; TCA, tricarboxylic acid; NO, nitric oxide; 1-MCP, 1-methyl cyclopropane; 6-BA, 6-benzyl amino purine; OA, oxalic acid; H_2_S, Hydrogen sulfide; BABA, β-aminobutyric acid; MT, melatonin.

Several treatments have shown positive effects on lowering PCI symptoms and maintaining cell energy status. Treatments upregulate the enzymes involved in oxidative phosphorylation, TCA cycle, and glycolysis (Fig. 3). Each treatment follows different mechanisms, including activating the antioxidant system, modulating the cell membrane stability, regulating respiratory rates, inducing heat shock proteins, and accumulating compatible solutes, thus enhancing cold tolerance by maintaining cell energy. Storage of produce at low-temperatures has been shown to delay senescence and preserve the postharvest quality of fruits, however, low-temperatures limit the shelf life of chilling-sensitive produce [49]. It has been reported that PCI is associated with an insufficient energy status of the produce, which initiates a loss of integrity of the cell membrane [20]. A decreasing trend in energy charge (EC) and ATP content has been reported with the onset of PCI. Decreased EC and ATP content contributed to decreased H^+^/Ca^2+^-ATPase activity, which in turn led to cell membrane instability. The activity of H^+^/Ca^2+^-ATPase is supposed to be crucial for the transport of ions across the membrane [50].

Application of methyl jasmonate [51], BABA [52], 1-MCP [53], glycine betaine [54], brassinosteroids [46], 6-BA [55], hydrogen sulfide [56], fibroin [57], trehalose [58], OA [59], NO [60], and MT [3], have been reported to attenuate PCI in stored produce by enhancing EC (Table 1). A study by Wang et al. [52] showed exogenous BABA on cold-stored cucumbers to upregulate the gene expressions of *CsATP*, *CsSDH*, *CsCCO*, *CsPCS*, and *CsOAT*. Elevated *CsATP*, *CsSDH*, and *CsCCO* expressions led to higher energy production, efficient electron transportation, enhanced respiration, and increased ROS scavenging. Higher expressions of *CsPCS* and *CsOAT* resulted in proline synthesis and improved stress tolerance.

**Table 1:** Role of treatments in promoting chilling tolerance in postharvest commodities during low-temperature storage

Crops	Treatment	Storage conditions	Effect with respect to control		Reference
			**Increase ↑**	**Decrease ↓**	
Apple	MT; 1 mM	1°C, 56 days	H^+^-ATPase	MDA, EL	[95]
Banana	EBL; 2.5 μM	7°C, 10 days	ATP, ADP, H^+^-ATPase, CCO, SDH	MDA, EL	[46]
	Fibroin; 1 g/L	6°C, 4 days	ATP, ADP, H^+^-ATPase, CCO, SDH		[109]
	NO; 0.05 mM	7°C, 20 days	ATP, ADP, H^+^-ATPase, CCO, SDH	MDA, EL	[60]
Cucumber	BABA; 10 mM	4°C, 3 weeks	ATP, ADP, H + -ATPase, CCO, SDH		[52]
	Hydrogen sulfide; 1.0 mM	4°C, 2 weeks	ATP, ADP, H^+^-ATPase, CCO, SDH		[11]
	6-BAP; 50 mM	2°C, 16 days	ATPase, Proline ATP, ADP	MDA, EL	[55]
	Trehalose; 200 mM	6–14°C, 2 weeks	ATPase	MDA, EL	[58]
Summer squash	1-MCP; 1 μL/L	0°C, 19 days	ATP, ADP, H^+^-ATPase, CCO, SDH	MDA, EL	[110]
Nectarine	1-MCP; 0.25 μL/L	0°C, 5 weeks	ATP, ADP, H^+^-ATPase, CCO, SDH		[53]
Mango	MT; 100 μM	5°C, 4 weeks	ATP, ADP, H^+^-ATPase, CCO, SDH		[38]
	OA; 5 mM	10°C, 49 days	ATP, ADP, H^+^-ATPase, CCO, SDH	MDA, EL	[59]
Papaya	GB; 15 mM	6°C, 40 days	ATP, ADP, H^+^-ATPase, CCO, SDH		[54]
Peach	24-EBL; 15 μM	5°C, 4 weeks	ATP, ADP, H^+^-ATPase, CCO, SDH		[111]
	OA; 5 mM	0°C, 35 days	ATP, ADP, H^+^-ATPase, CCO, SDH		[18]
	MeJA; 1 μM	0°C, 5 weeks	ATP, ADP, H^+^-ATPase, CCO, SDH		[51]
Pear	MeJA; 10 μM	3°C, 16 weeks	ATP, ADP H^+^-ATPase, CCO, SDH	MDA, EL	[112]
	1-MCP; 0.5 ml/L	0°C, 90 days	ATP, ADP H^+^-ATPase, CCO, SDH	MDA, EL	[86]
Tomato	MT; 100 μM	4°C, 28 days	ATP, ADP H^+^-ATPase, CCO, SDH	MDA, EL	[113]

ADP: Adenosine diphosphate; AMP: Adenosine monophosphate; ATP: Adenosine triphosphate; BABA: β-aminobutyric acid; CCO: Cytochrome c oxidase; EBL: Epibrassinolide; EL: Electrolyte leakage; GB: Glycine betaine; MDA: Malondialdehyde; MeJA: Methyl jasmonic acid; MT: Melatonin; NO: Nitric oxide; OA: Oxalic acid; SDH: Succinate dehydrogenase; POX: Peroxidase; SOD: Superoxide dismutase; 1-MCP: 1-Methylcyclopropene

Methyl jasmonate, an endogenous signaling molecule, has been reported to play an important role in attenuating environmental stress and promoting fruit ripening, plant growth, and development. Methyl jasmonate was successful in controlling the effects of PCI in peaches [61], tomatoes [62], and loquat [43]. H^+^-ATPase, CCO, Ca^2+^-ATPase, and SDH are the enzymes responsible for energy metabolism. ATPases are enzymes which by catalyzing the breakdown of ATP, lead to the formation of ADP and a free phosphate. The H^+^-ATPase present in the plasma membrane ensures that the protons are pumped out of the cell into the lumen of the vacuole. This creates a pH difference across the cell membrane. Succinate is oxidized to fumarate by SDH, which leads to ATP production in mitochondria. The activity of the H^+^-ATPase in papaya is considered a marker for ripening and senescence [63].

Treatment of peaches with methyl jasmonate showed higher H^+^-ATPase, CCO, Ca^2+^-ATPase, and SDH activities along with high EC and ATP levels, suggesting that boosting these enzymes with methyl jasmonate application improves cold tolerance in peaches [51]. It has also been documented that methyl jasmonate in tomato and loquat regulates the catabolism of arginine which enhances the aminobutyric acid and proline content and thus increases chilling tolerance [43]. The H_2_S-treated cucumbers showed higher activities of H^+^-ATPase, CCO, Ca^2+^-ATPase, and SDH enzymes, which are considered essential for facilitating energy metabolism. The activity trend of these enzymes reflects mitochondrial function and their ability to synthesize energy [18]. Shifts in SDH and CCO activities affect the activity of the ETC, which in turn affects energy status [14]. Any change or deactivation of energy-metabolizing enzymes leads to mitochondrial dysfunction, followed by insufficient energy production and, thus, cell death. Therefore, PCI is associated with energy deficiency in plants. H_2_S-treated, cold-stored bananas and cucumbers showed higher H^+^-ATPase, CCO, Ca^2+^-ATPase, and SDH activities, which helped maintain higher levels of ATP and EC and thus inhibit PCI [18, 64].

Sugar indirectly contributes to a great source of energy. The glycolysis and pyruvate decarboxylation breaks down sugars into acetyl-CoA, which enters the TCA cycle [65]. The TCA cycle utilizes acetyl-CoA and produces ATP, NADH, and FADH_2_. Further, NADH and FADH_2_ donate their electrons to ETC, producing ATP via anaerobic processes [66]. Thus, by providing readable available energy, sugars supply helps in mitigating chilling stress and disrupting cellular metabolism. PCI may lead to severe water loss via a damaged membrane [67]. Sugars such as sucrose behave as osmolytes and play an essential part in maintaining cellular water balance, thus providing strength to fruit to cope with the chilling stress [68]. Thus, the PCI in plants is closely associated with sugar metabolism.

Another essential element in mitigating PCI by providing sufficient ATP supply is fibroin. Fibroin is a natural protein that maintains cell membrane integrity in harvested fruits and vegetables by modulating water vapor diffusion and gas exchange [57]. It has been documented that fibroin-treated bananas stored at 6°C showed a delayed respiration rate, thereby delaying the consumption of ATP. Fibroin was successful in differentially expressing energy-related genes that increased ATP levels and, in turn, maintained cell membrane integrity. Cell membrane potential has been linked to lowering the electrolyte leakage and thus improved chilling tolerance of harvested products [57].

Soluble sugar levels after fibroin treatment are supposed to increase water-holding capacity and thus reduce the dehydration stress caused by PCI. Moreover, sugars are involved in carbon and energy metabolism by serving as substrates for respiration [69]. Similarly, trehalose also served as a great source of energy and protects plants from PCI. Application of trehalose to guava increased membrane permeability in guava, reduced the MDA content and lowered the electrolyte leakage. In addition, it increased the enzymatic activities of Ca^2+^-ATPase and H^+^-ATPase, which maintained higher energy levels and helped in tolerating chilling stress [58].

The OA is a ubiquitous natural organic acid found in organisms, mainly plants and has been reported to increase energy status in harvested produce. The OA-treated cold-stored mangoes have higher ATP and EC levels due to better membrane integrity and proline accumulation [59]. The NO has been considered a key signaling molecule that acts in response to biotic and abiotic stress. The NO has been credited with suppressing ATP synthesis in plant mitochondria by inhibiting the cytochrome pathway [70]. Cold-stored bananas treated with NO were successful in maintaining energy metabolism to carry out the process of glycolysis, OPP, and TCA. This increased glucokinase and fructokinase, which enhanced the levels that maintained sufficient energy levels to combat PCI [71].

A non-protein amino acid, known as BABA, is a xenobiotic molecule capable of enhancing the phylactic power of plants against stress conditions [72]. Cucumbers treated with BABA showed higher H^+^-ATPase, COO, Ca^2+^-ATPase, and SDH enzyme activities during cold storage, thereby maintaining higher levels of ATP and ADP. Thus, BABA treatment in produce contributed to the reduction of PCI by maintaining higher energy status [11]. 1-MCP, an ethylene inhibitor, reacts with ethylene receptors and is widely used to extend the shelf life of many harvested fruits, including cold-stored peaches [73] and red pitaya [74]. It also follows the above mechanism of maintaining higher energy levels.

Brassinosteroids are hormonal sterols that are widely distributed in plant tissues. They play an important role in plant development. A higher ratio of NADH/NAD^+^ was observed in EBR-treated, cold-stored banana, indicating high energy production. ROS burst was inhibited by the activation of SDH and CCO enzymes, preventing oxidative damage [46]. Abscisic acid is a key phytohormone that, when applied to ‘Natura’ and ‘Sinatra’ varieties of zucchini stored at 4°C, increases their chilling tolerance by decreasing the MDA levels, electrolyte leakage [75], and regulating phenolic metabolism and non-enzymatic antioxidant system [76]. Benzyladsenine, also known as 6-BA or BAP, is a cytokinin known to induce plant growth and development and cell growth stimulation. It is known to inhibit the degradation of chlorophyll, thereby delaying senescence. Cucumbers treated with 50mM L^−1^ BAP resulted in higher levels of ADP, ATP, and EC and thus reduced PCI [55].

It has been reported that MT-treated mangoes stored at 5 ± 1°C showed a great response in alleviating PCI. The NO is a major signaling molecule that, when applied to zucchini stored at 4°C, reduced the weight loss by lowering the metabolic activity, transpiration rate and electrolyte leakage, and therefore maintained the membrane integrity. Moreover, enhanced SOD and CAT activity leads to the mitigation of PCI [77]. Preconditioning was found to be beneficial in mitigating cold stress in zucchini stored at 4°C, by enhancing the CAT and SOD enzymatic activity [78]. Zucchini stored at 4°C was reported to be tolerant against PCI due to increased levels of glucose, fructose, and pinitol [79].

Application of MT to mangoes has demonstrated higher H^+^-ATPase, COO, Ca^2+^-ATPase, and SDH enzyme activity, followed by enhanced iATP production, thereby maintaining cell membrane integrity [3]. Similarly, methyl SA and methyl jasmonate treatments are also known to maintain membrane integrity in pomegranate through increased ATP accumulation, which contributes to increased chilling tolerance [80]. MT treatment alleviated chilling stress in cold-stored eggplants by reducing ROS, MDA, and EL levels. These reductions were due to increased activities of SOD, CAT, endogenous polyamines, and MT. A 100μM MT concentration interacted with the transcription factor and regulatory proteins and effectively enhanced the expressions of *SOD* and *CAT1/2*. Additionally, MT enhanced the expression of genes involved in polyamine synthesis (*ADC* and *ODC*), MT synthesis (*TDC, T5H, SNAT, ASMT,* and *COMT*), and cold regulation (*COR1, CBFa/b,* and *ZAT2/6/12*) [81]. Similar results were found in cold-stored eggplants (10°C for 2 days) subjected to LTC [82].

### Energy status in attenuating postharvest chilling injury

The ATP is the primary requirement for ionic active transportation across cell membranes. They are essential for maintaining membrane integrity by balancing calcium and potassium ions. Many enzymatic reactions have ATP as an essential cofactor. The PCI leads to protein disruption and protein denaturation. The ATP levels determine protein synthesis and repair mechanisms, elevating the defense mechanisms against PCI [83]. ATP is responsible for regenerating GSH (glutathione), an essential antioxidant. Thus, ATP helps in ROS scavenging and protecting fruits against PCI. Chilling stress is known to accelerate cell membrane transition, converting it from a fluid liquid-crystalline to a rigid solid gel, marking increased electrolyte leakage, and interrupting the activity of iATP supplying enzymes, leading to a deficiency in iATP.

The iATP shortage is followed by the enhanced enzymatic activity of phospholipase D (PLD) and lipoxygenase (LOX), accompanied by increased accumulation of ROS. These increased PLD and LOX levels result from impaired Ca^2 + -^ATPase activity, leading to increased cytosolic Ca^2+^ [84]. In addition, the activity of NADH dehydrogenase (complex I) and cytochrome b/c1 oxidoreductases (complex III) is interrupted, leading to increased ROS accumulation. Enhanced enzymatic activity of LOX and PLD, with concomitant increased accumulation of ROS leads to loss of membrane integrity accompanied by peroxidation of unSFA present in the membrane, which is characterized by higher accumulation of MDA [84].

The MDA accumulation and electrolyte leakage have been of great value in the study of membrane integrity, as they have been considered reliable markers for membrane unSFA peroxidation and cell membrane semi-permeability losses, respectively [26]. Exogenous OA to tomatoes stored at 4 ± 0.5°C for 20 days alleviated CI development and membrane damage by lowering electrolyte leakage and MDA accumulation. OA increased the levels of H^+^-ATPase and Ca^2+^-ATPase. Additionally, OA upregulated the expressions of *PSY1* and *ZDS* genes through a combination of signal transduction activation, transcription factor regulation, epigenetic modifications, and metabolic adjustments. The enhanced expressions of *PSY1* and *ZDS* led to enhanced lycopene accumulation and attributed good quality to tomatoes [85].

Attenuation of PCI by application of 6-BA in cucumber [55], OA in peaches [18], 1-MCP in pears [86], NO in banana [35], glycine betaine in peaches [87], hydrogen sulfide in bananas [64], low-temperature conditioning in loquat [88], methyl jasmonate in peaches [51], and MT in mango fruit [38], are due to adequate iATP availability, which reduces electrolyte leakage and MDA accumulation, thus maintaining membrane integrity.

It has been reported that the PCI in blueberry stored at 0°C for 60 days, manifested by browning and external pitting, experiences iATP shortage, which is due to a reduction in the enzymatic activities of H^+^-ATPase, Ca^2+^-ATPase, CCO, and SDH, leading to a reduction in ATP and ADP levels, increased AMP levels, leading to decreased AEC levels [89]. Banana stored at 7°C for 30 days experienced PCI followed by insufficient iATP, decreased ATP and ADP, and enhanced AMP resulting in decreased AEC, leading to increased activity of LOX and PLD enzymes, accelerating fruit senescence.

Papaya stored at 1°C showed a higher PCI index than those stored at 6°C and 11°C, and was accompanied by the increased enzymatic activity of H^+^-ATPase, Ca^2+^-ATPase, CCO, and SDH. This led to an increase in iATP supply, decreased ATP and ADP, and enhanced AMP leading to decreased AEC [29]. Ion leakage in zucchini has been regarded as a marker of degradation. The electrolyte leakage has been reported to increase in zucchini stored at 2.5°C, as compared to the ones stored at 4°C [90]. It experienced higher H_2_O_2_ accumulation, leading to higher ROS accumulation. A study by Palma et al. [91], conducted on two zucchini varieties ‘Natura’ and ‘Sinatra’ concluded that putrescine is accumulated in both varieties at low-temperatures which enhances the GABA shunt pathway and helps to maintain energy. As the stress becomes severe, ornithine decarboxylase activity is enhanced. This increases the proline levels and contributes to mitigating the chilling stress. A similar study conducted by [92] concluded that putrescine increases the antioxidant response and carbohydrate content in zucchini.

A sufficient and continuous iATP supply is considered essential for the conversion of acetyl-CoA carboxylase (ACCase) into membrane fatty acids. The biosynthesis of fatty acids is crucial for the conversion of acetyl-CoA to produce malonyl-CoA, which plays its role in restoring membrane fluidity due to chilling stress [93]. Banana treated with NO (0.05 mM) stored at 7°C for 20 days attenuated chilling stress for a similar reason mentioned above. This was accompanied by higher levels of fructokinase and glucokinase, and G6PDH along with 6PGDH, revealing an increased activity of the EMP and OPP pathways, respectively. This resulted in less electrolyte leakage and higher membrane integrity [60].

Pears stored at 0°C for 180 days experienced peel browning accompanied by insufficient iATP, lower ATP, ADP, AEC, and increased AMP. Moreover, the molecular studies revealed decreased gene expression levels of *ATPase*, *NADH dehydrogenase* (*NDA*), and *vacuolar proton-in-organic-pyrophosphatase* (*VPP*) and increased gene expression levels of *PLD*. Decreased gene expression levels of *ATPase* led to metabolic dysfunction, membrane potential disruption, and energy deficiency. Lower expressions of *NDA* and *VPP* genes resulted in reduced electron transport, increased ROS production, impaired respiration, and disrupted vacuolar functions. Higher *PLD* expressions resulted in the breakdown of membrane phospholipids into phosphatidic acid and free fatty acids, leading to membrane destabilization and degradation [94].

Exogenous 1 mM MT to apples stored for 56 days at 1°C upregulated genes such as *DNA-damage-repair/ toleration protein 100* (*MdDRT100*, *MD11G1028200*), *senescence-associated carboxylesterase 101* (*MdSC101, MD09G1038700*), *low-temperature-induced protein 65* (*MdLTI65, MD07G1268800*), and *17.3 kDa class I heat shock protein 11* (*MdHSP11*, *MD17G1020300*). These results indicated that MT inhibits ethylene biosynthesizing genes, maintains energy levels, and induces the expression of stress-resistant genes [95]. A 1 mM dose of glycine betaine to sweet peppers stored at 3°C reduced PCI by elevating the expression levels of SOD, APX, and CAT genes. The increment in the enzymatic activities as well as transcription levels of SOD, APX, and CAT, act as ROS scavengers that protect the mitochondria from damage and maintain continuous ATP supply [96].

Low-temperature conditioning (10°C for 25 days) attenuated PCI through higher iATP, lower ATP, ADP, AEC, and increased AMP. This has been attributed to the higher enzymatic activity of V-ATPase, NADH dehydrogenase, and ATP synthase, contributing to lower electrolyte leakage and higher membrane integrity [97]. Ensuring sufficient and continuous availability of iATP is essential for heat shock proteins (HSPs), which are documented to mitigate chilling stress and fungal decay in horticultural produce. HSPs consist of domains with N-terminal ATP binding sites, which are important for them to perform their biological functions [98]. The HSPs have free amino acid residues that enable them to exhibit ROS scavenging activity, and are known to have a synergistic relationship with the antioxidant system. Increased antioxidant activity helps them to boost the GSH/GSSG and AA/DHA ratio, which helps in maintaining postharvest nutritional quality [99].

**Figure 4:**
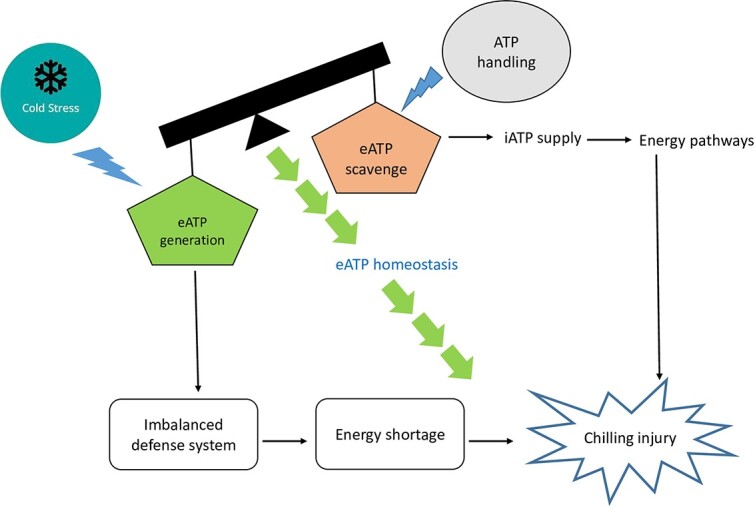
Role of ATP homeostasis in mitigating chilling injury of postharvest commodities. A right balance between eATP generation and sufficient eATP scavenging is essential for generating sufficient iATP levels which may elevate energy pathways including, EMP, PPP, and TCA to mitigate PCI. Abbreviations: eATP, external adenosine triphosphate; iATP, internal adenosine triphosphate; EMP, Embden–Meyerhof–Parnas pathway; PCI, postharvest chilling injury; PPP, pentose phosphate pathway; TCA, tricarboxylic acid.

The HSPs have been documented to inhibit the enzymatic activity of phospholipase, as they are responsible for binding membrane phospholipids. This leads to decreased peroxidation of the unSFA membrane, ROS accumulation, and membrane degradation due to decreased PLD enzymatic activity [100]. ATP homeostasis aids in mitigating CI in postharvest commodities by maintaining a right balance between ATP scavenging and ATP generation (Fig. 4). Apyrase as a supplier of Ca^2+^ and Mg^2+^ has been studied to maintain extracellular ATP (eATP) homeostasis, which is essential to combat biotic and abiotic stress [84]. Chilling stress in *Populus euphratica* is known to disrupt cell membrane integrity, which leads to electron expulsion from its cytoplasm to the apoplast, resulting in generation of insufficient eATP, ultimately leading to the disruption of membrane repair. This enhances electrolyte leakage by reducing membrane integrity [101]. Overexpression of the apyrase 2 (*PeAPY2*) gene in the *Arabidopsis* plant leads to eATP hydrolysis, which helps to combat the chilling stress by exhibiting reduced electrolyte leakage. It helps in the generation of sufficient eATP, which promotes vesicular transport leading to improved membrane integrity. Therefore, eATP is considered essential for maintaining membrane integrity.

### Potential of NAD in mitigating postharvest chilling injury

NAD is essential in several biological activities that help mitigate PCI in fruits [24]. NAD+ is crucial for glycolysis, acting as an electron carrier and converting glucose into pyruvate. In the TCA cycle, NAD+ is reduced to NADH as it assists in oxidizing acetyl-CoA. These activities are vital for generating ATP, providing energy for cellular functions, and mitigating stress during PCI [102]. Additionally, NADH donates electrons to the ETC in the mitochondria, driving ATP production via oxidative phosphorylation. Efficient ATP generation maintains cellular integrity and metabolic functions during PCI [25].

The onset of PCI results in enhanced ROS generation. In tomatoes, for example, NADH provides reducing power for regenerating antioxidants like glutathione and ascorbate, which neutralize ROS and lower oxidative damage [103]. NAD+ is a cofactor for various antioxidant enzymes like glutathione reductase, which reduces oxidized glutathione and maintains redox homeostasis. NAD+ also synthesizes polyamines, including spermidine and spermine. These compounds stabilize cell membranes, scavenge ROS, regulate ion channels, and help mitigate PCI [16]. In addition, polyamines also influence the expressions of stress-responsive genes, improving tomatoes’ ability to combat PCI. Moreover, the phosphorylated NAD, NAD+, and NADPH forms are essential for calcium signaling. This calcium signaling mediated by NAD-related pathways helps maintain cellular homeostasis, which is necessary for PCI mitigation in tomatoes [104]. Furthermore, NAD+ supports reactions that replenish TCA cycle intermediates, ensuring an uninterrupted supply of energy production and biosynthesis processes [105]. This metabolic flexibility maintains cellular functions and helps in PCI mitigation.

### Role of eATP and its receptor *DORN1* in mitigating chilling stress

The perception of eATP by receptor kinase *DORN1* plays a significant role in mediating cold stress responses. eATP is recognized as an essential plant signaling molecule involved in multiple physiological processes [106]. A study by Shan et al. [107] reported 1 mM ATP to mitigate PCI by upregulating *MaDORN1* in bananas stored at 6°C. *DORN1* is a receptor kinase localized in the plasma membrane that binds to eATP. This interaction triggers a cascade of intracellular signaling events. As eATP levels increase, *DORN1* binds to eATP, initiating a signaling cascade. This binding activates the kinase domain of *DORN1*, leading to the phosphorylation of downstream targets and activation of secondary messengers like Ca^2+^ ions and ROS [108]. This activation results in the regulation of gene expressions involved in PCI-stress responses. PCI leads to ROS accumulation, which causes oxidative stress.

Activation of *DORN1* by eATP results in enhanced activity of CAT and SOD, which scavenge ROS and protect the plants from oxidative stress. Additionally, eATP perception by *DORN1* leads to enhanced cytosolic Ca^2+^ ions, which activate calcium-dependent protein kinases (*CDPK*s), phosphorylate the target proteins, and mitigate PCI [106]. Thus, eATP perception by *DORN1* plays a vital role in combating PCI by elevating antioxidant levels, regulating gene expressions, stabilizing membrane integrity, modulating calcium signaling, and maintaining cellular metabolism.

## Conclusion and future perspectives

The PCI leads to significant economic losses. The ADP/ATP carriers are essential for transportation of ADP into mitochondrial matrix and ATP outside the mitochondria to maintain a high ATP concentration in the cytosol, being important for performing energy requiring processes. The ATP is an essential requirement in respiratory pathways for converting the inactive forms into active forms. Thus, the biosynthesis of ATP at the intracellular level is the result of the EMP, TCA cycle, OPPP, the arginine pathway, the cytochrome oxidase pathway, and the GABA shunt pathway. These pathways ensure proper energy supply essential for providing capability to cope with the chilling stress. Postharvest chemical treatments are widely accepted for their ability to mitigate chilling stress by accumulating GABA. Irrespective of the available work, there is a need for elucidation of the interconnected mechanisms of eATP activation, eATP production, and signaling of ROS molecules.

This may aid in elucidating adaptive measures against stress responses, including oxidative stress, microbial attack, and temperature fluctuations. Identifying critical genes responsible for eATP production and iATP generation can help develop treatments to mitigate PCI. An idea of these interconnected mechanisms might be useful in optimizing storage and transportation of fruits. Certain technological advancements can also aid in better understanding of PCI in fruits. Non-destructive imaging techniques may be employed for determining PCI severity. Transcriptomics, proteomics, and metabolomics can be employed to identify genetic responses, metabolites involved, and proteins affected during PCI. Data analytics and machine learning can also be employed to develop patterns, correlations, and models for optimizing PCI management strategies. Further research requires attention to the signaling molecules and eATP interplay to narrow down to several biochemical reactions and respiratory/physiological pathways to mitigate PCI in fruits during cold storage.

## Supplementary Material

Web_Material_uhae204

## Data Availability

No data was used for the research described in the article.
