# Supplementary material for: Is ATP a signaling regulator for postharvest chilling tolerance in fruits?
Source: Hortic Res. 2024 Jul 26;11(9):uhae204. doi: 10.1093/hr/uhae204 (PMC11404123; doi:10.1093/hr/uhae204)
Supplement: Web_Material_uhae204 [file web_material_uhae204.zip › Supplementary material-HR.docx]

# Economic loss due to postharvest chilling injury in fruits

Understanding postharvest chilling injury (PCI) is essential in postharvest technology as it is a marker for quality preservation, shelf-life extension, marketability, consumer satisfaction, and economic considerations [1]. PCI is considered a physiological dysfunction in some sensitive crops maintained at non-freezing but low, non-tolerable temperatures for an extended period. Understanding PCI may result in the correct identification of its causative factors. Implementing appropriate postharvest practices can aid in extending the shelf-life of produce. Elucidating the PCI mechanisms may enhance the development of cost-effective strategies to minimize its occurrence [2]. This can increase profitability and create a more efficient and sustainable supply chain.

The PCI can occur in horticultural produce at any stage of the supply chain, including harvesting, storage, transportation, and distribution [3]. Once removed from the chilling temperatures, the stored fresh produce will recover and function normally after a short exposure to chilling stress. Unfortunately, prolonged exposure to chilling temperature leads to irreversible metabolic imbalances. The occurrence and severity of PCI signs depend on various factors like cultivar, species, environment, duration of exposure, metabolic status, and tissue type [4]. Postharvest chilling injury can occur in fruits and vegetables when stored at low temperatures, but signs of PCI become apparent after the produce is moved to ambient temperature (Table 1).

The PCI has been reported to have adverse effects on quality. It has been associated with various metabolic disorders, including skin pitting, aroma and flavor loss, ripening inhibition, improper softening, and surface discoloration. Fruit-specific PCI symptoms may develop, including discoloration in tomatoes [5], mealiness and wooliness in peaches and nectarines [6], superficial scald in apples [7], flesh browning in pineapples [8], skin streaks in bananas [9], skin lignification in kiwifruit [10], pulp browning in eggplant [11], and lesions in cucumbers [12].

Although PCI can result in significant postharvest loss and waste, it becomes challenging as it is difficult to identify the exact point in the supply chain at which it occurs. PCI can be compounded by field chilling, chilling during transit, and refrigeration at home, which can lead to PCI in the produce. Maximum damage is often observed in retail outlets or at the consumer's end, making monitoring even more difficult. Furthermore, PCI signs are internal in many species, like nectarines and pineapples [13], making any abnormality in flavor and texture detectable only upon consumption. Signs of PCI are often misdiagnosed; for example, increased susceptibility to pathogens from PCI is often mistaken as the main reason for poor quality and loss of postharvest produce, which can be due to poor selection of the variety or early harvesting. Because of these factors make quantifying economic losses due to PCI challenging [12].

In 2018, the global trade of fresh fruits and vegetables accounted for US$ 115 billion. It has been reported that among the top 50 commodities traded globally, more than 50 % were susceptible to PCI. Postharvest losses and waste have been estimated at around 33 % globally; even an increase in PCI by 0.5 % of postharvest losses and waste would represent economic losses of US$ 144 million. [14]. For example, a commonly traded commodity like citrus reports 25% postharvest losses due to PCI. It is also to be noted that the cost of shipping at a temperature above 4 °C (commonly used) and the complex logistics have yet to be included while reporting losses in fruits due to PCI [15]. Periodic storage at inappropriate temperatures to extend the product's shelf life may outweigh the negative impact on quality in some cases, for example, if the fruits are stored at temperatures that cannot cause PCI and are rejected due to spoilage. In contrast, there may be sellable fruits as the PCI sign is invisible. This encourages the refrigeration of perishables and chilling-sensitive produce, which may be profitable in the short term but may lead to long-term consequences in terms of produce value and quality [16, 17].

**Table 1.** Major PCI signs due to inappropriate storage temperature.

| **Fruit** | **Minimum safe temperature (°C)** | **Time duration (week)** | **Observed PCI signs** | **Reference paper** |
| --- | --- | --- | --- | --- |
| Apple | 0–2 | 1-2 | Pitting, off-flavor | [41] |
| Banana | 13 | 12 d | Delayed yellowing | [42] |
| Carambola | 10 | 3 | Dark ribs, skin discoloration, skin browning | [43] |
| Cucumber | 7 | 1 | Surface pitting, internal discoloration, tissue collapse, pitting, translucent water-soaked spots | [12, 44] |
| Guava | 5 | 2 | Fruit decay, surface breakdown | [45] |
| Kiwifruit | 0 | 24 | Skin lignification | [10, 46] |
| Lemon | 10 | 3 | Rind staining, pitting, red blotches, scald, and watery breakdown | [47] |
| Loquat | 1-4 | 3 | Poor flavor, less juice, wrinkled peel, stuck peel, firm and juiceless texture, internal browning | [48, 49] |
| Mango | 10-13 | 2-4 | Pulp discoloration, a water-soaked appearance, internal browning | [50-53] |
| Mandarins | 10 | 1 | Weight loss, necrotic and sunken areas on the peel | [54] |
| Orange | 5 | 2 | Pitting, browning | [55, 56] |
| Peach | 5 | 1-2 | Leatheriness, dry, mealy, woolly, graininess, flesh browning, flavor loss | [57-59] |
| Pear | 0 | 4 | Peel browning spots, ripening and senescence scalding | [60, 61] |
| Papaya | 6-12 | 3 | Skin pitting, scald, external water soaking | [62] |
| Pineapple | 7-12 | 2-3 | Internal browning, pitting on the outer peel surface | [63] |
| Plum | 8 | 1-2 | Loss of flavor, black pit cavity | [64] |
| Pomegranate | 7 | 4 | Skin and internal browning | [65, 66] |
| Sapota | 14 | 2 | Dark-brown spots and pitting of the skin | [67] |
| Tomato | 10-13 | 2-3 | Water-soaked appearance, internal breakdown, and failure to ripen, off-flavour, blotchy coloration, and decay | [68] |

PCI: Postharvest chilling injury

# Mechanisms of postharvest chilling injury

Many scientists believe in a universal mechanism for PCI in the plant kingdom [18]. Although this is a controversial point, it has not been conclusively disapproved. The first theory was offered by Lyons in 1973. Lyons’ theory highlighted the bulk membrane lipid phase transition into the gel phase lipids at a critical temperature and the conversion of the lipid phase into the gel phase due to cold temperature, leading to leakage of cellular membrane contents, resulting in integrity loss. This transition ultimately results in an irreversible metabolic imbalance, leading to plant death. In support of this theory, a piece of early evidence was based on the plant mitochondrial study [19]. A greater degree of lipid unsaturation was recorded in chilling-resistant plants compared to chilling-sensitive ones. This resulted in speculation of a relationship between chilling sensitivity and the physical structure of cellular membranes [20]. The theory suggests dividing the occurrence of PCI into two phases. The first phase is a temperature-dependent phase, which is also regarded to be a primary event. These are known as primary events, as their initiation starts as soon as the storage temperature reaches below the threshold temperature for a prolonged duration. The metabolic dysfunctions caused by these primary events lead to certain secondary events, which develop based on the duration of exposure to the chilling temperature [18].

The damage to the cell membrane hypothesis and reactive oxygen stress hypothesis are the two hypotheses proposed in fruits based on the underlying biochemical mechanisms [21]. Many studies have reported damage to the inner cellular membrane to be the primitive event of PCI in fruits. This cellular damage leads to various subsequent physiological and metabolic dysfunctions in fruits, accompanied by several catalytic reactions [22]. With the onset of PCI in fruits, low-temperature stress leads to the structural conversion of the cell membrane from a flexible liquid crystalline structure to a gel state. This results in the depletion of the unsaturated fatty acids, causing functional damage to the membrane. The PCI also results in metabolic disorders, such as insufficient energy supply, the primary secondary metabolic dysfunction found in the chilling-affected fruits. The second hypothesis deals with the effects of a burst of ROS in fruits [23]. Chilling stress leads to the excessive accumulation of ROS, which includes hydrogen peroxide (H_2_O_2_), singlet oxygen (^1^O_2_), hydroxyl radical (HO^.−^), and superoxide radical (O^2−^). These species can damage the cellular membrane by oxidizing the fatty acids, proteins, and nucleic acids [23]. As the theories stated on PCI revolve around a standard feature, these are considered converging rather than diverging. This makes it all the more difficult to view the loss of cellular integrity, dysfunction of membrane-based enzymes, and membrane lipid domains.

Several areas of PCI concerning adenosine triphosphate (ATP)’s role still need further investigation. These include ATP depletion and energy metabolism, membrane integrity and ATPase activity, ROS production and antioxidant defense system, calcium signaling and ATP involvement, and genetic and molecular regulation. As PCI is documented to be linked with a reduction in ATP levels, further understanding the biochemical pathways involved might be helpful [24]. Investigating the relationship between ATP levels, ATPase activity, and membrane dynamics may provide helpful information about PCI [25]. More studies on the interplay between ROS production, ATP levels, and antioxidant enzymatic and non-enzymatic defense systems may facilitate the development of potential PCI mitigation strategies [26].

Additionally, the genes related to ATP-bound processes during PCI may lead to finding the critical genes affected in PCI-affected fruits. Further research is therefore needed to draw a sequential explanation of PCI. Only when the mechanisms of PCI are precise can the efforts to ameliorate this problem be successful.

# Role of eATP in iATP salvaging to mitigate postharvest chilling injury

The intracellular ATP (iATP) salvaging refers to the process by which extracellular ATP (eATP) saves iATP from destruction. The eATP plays a significant role in maintaining homeostasis for iATP salvaging. The process of iATP salvaging by eATP is still an evolving area that requires significant research [27, 28]. However, few mechanisms contributing to iATP salvaging have been proposed. One mechanism documented believes specific transporters present on the plasma membrane of fruits possess an affinity for eATP, thus facilitating its uptake followed by its conversion to iATP via ectonucleotidases. Endonucleotidases are enzymes on the cell surface capable of hydrolyzing eATP into ADP and inorganic phosphate (Pi). This ADP can again be converted to ATP by subsequent enzymatic reactions [25].

Another proposed mechanism focuses on the redistribution of eATP within the cell. It states that eATP diffuses across the cell membrane of fruits to the intracellular cell compartments, where it is salvaged to regenerate iATP. This regenerated iATP helps maintain the fruit's membrane potential, facilitating metabolic reactions, sustaining protein synthesis, and driving active transport mechanisms [29]. Thus, iATP provides energy essential for cell survival and recovery and provides energy and maintains cell homeostasis during cold stress [30]. These mechanisms require further investigation as they might be fruit-specific, and the reactions might vary. Additionally, the duration and severity of PCI might lead to variations in salvaging mechanisms. To elevate chilling tolerance, more studies are required to identify specific enzymes, transporters, and intracellular pathways involved in iATP salvaging.

## **Methods for eATP detection**

The eATP detection is of utmost importance in the context of PCI. Appropriate detection and quantification of eATP may help understand its signaling role [31]. Moreover, it may help assess cellular energy status. The PCI in fruit cells may result in a drastic decline in ATP levels [32]. Properly monitoring eATP levels may be used to estimate its availability, which may be utilized in the fruits' cells for replenishing iATP. This detection can also help researchers to evaluate the potential of ATP salvaging mechanisms. The chilling-affected fruits are associated with impaired ATP production [21]. Detection of eATP can be used for identifying potential PCI markers [25]. An increase or decrease may denote the severity of PCI on the energy metabolism. Understanding the dynamics of eATP levels with PCI may contribute to improved fruit quality and extended shelf-life.

A series of biological activities responsible for maintaining energy metabolism and cell signaling is governed by iATP, whereas eATP mediates the initial signal for triggering downstream processes in plant cells. Thus, extracting and separating eATP from iATP is essential to prevent contamination. To elucidate the role of eATP in plants, its determination becomes essential (Table 2). Cold-stressed fruits experience damaged cell membranes, leading to ATP leakage. The amount of leakage of eATP from the fruit cells governs the quantitative analysis of eATP concentrations [25]. The liquid culture media collected from fruits under stress conditions can be used to detect ATP leakage. The amount of ATP leakage under stress conditions governs the extent of fruit membrane damage affecting the permeability. Low-temperature storage of fruits results in the initiation of various physiological activities; thus, investigating eATP leakage becomes essential to elucidate PCI occurrence.

Various approaches have been developed to date for ATP quantification. Nuclear magnetic resonance (NMR) spectroscopy [33], high-performance liquid chromatography (HPLC) [34], and luciferase methodology [35] are a few traditional methods applied for ATP determination. A luciferase assay at pM and nM levels was applied for ATP quantification in *Arabidopsis* leaves and *Picea meyeri*, respectively [36]. Developing a cellulose-binding domain (CBD)-luciferase technique clearly understood the eATP distribution in *Medicago truncatula* at the μM level [31].

The fluorescence technique proved excellent and sensitive, with a diverse linear range from 8.0 pM to 4.0 mM [37]. However, optical decomposition and contamination are some of the fluorescence technique's shortcomings. Due to their high specificity and affinity, aptasensors are an excellent alternative for monitoring eATP [38]. The chemical stability and design flexibility of aptasensors makes them superficial over other traditional affinity reagents. These beneficial features have made it possible to use electrochemical, calorimetric, and fluorescent sensors for eATP monitoring [39]. Recently, the use of gold nanoparticles (AuNPs) in calorimetric biosensors has been seen due to their ultrahigh extinction coefficient. An excellent analytical ATP determination is exhibited by electrochemiluminescence aptasensors between the concentration range of 0.5 pM and 1.0 mM [40]. Therefore, it can accomplish the requirements of the ATP determination limit well and can be successfully used for monitoring eATP in fruits during postharvest operations.

**Table 2.** Methods for determination of ATP.

| **ATP detection methods** | **Limit** | **Range of dissociation constant** | **Benefits** | **Reference paper** |
| --- | --- | --- | --- | --- |
| NMR spectroscopy | 2.9 nM | 0.2−0.5 μM | - Non-radiation tool - Sensitive to ATP | [33] |
| Luciferase methodology | 0.2 nM | 0.010−10 mM | - Faster and more sensitive - Suitable for different cell types | [35] |
| HPLC | 617.6 nM | 2.09−269 μM | - Simple - Useful in studying energy metabolism | [34] |
| Peptide/Au NCs and hemin-G-quadruplex DNAzyme based Colorimetric aptasensor | 0.00135 nM | 0.01−1 nM | - Excellent selectivity for ATP | [69] |
| ATP binding aptamer-based on unmodified AuNPs | 50 nM | 50−1000 nM | - Highly sensitive and selective for ATP calorimetric detection | [70] |
| Carbon nanoparticles-Fe^3+^ based fluorescent aptasensor | 0.48 μM | 0.5−50 μM | - Sensitive and selective at detecting ATP | [71] |
| DNA-Ag nanoclusters fluorescence light-up aptasensor | 28 μM | 50−100 μM | - Simple and cheap operation - Label-free fluorescence strategy - Requires only small changes in the sensing sequence | [72] |
| Hairpin aptamer probe colorimetric aptasensor | 100 nM | 0−500 nM | - Simple, rapid, and cost-effective | [73] |

ATP: Adenosine triphosphate; DNA: Deoxyribonucleic acid; HPLC: High-performance liquid chromatography; NMR: Nuclear Magnetic Resonance

**References**

1. Rai A, Kumari K, Vashistha P. Umbrella review on chilling injuries: Post-harvest issue, cause, and treatment in tomato. *Sci. Hortic.* 2022**;293**:110710. <10.1016/j.scienta.2021.110710>
2. Tan GH, Ali A, Siddiqui Y. Current strategies, perspectives and challenges in management and control of postharvest diseases of papaya. *Sci. Hortic.* 2022;**301**:111139. <10.1016/j.scienta.2022.111139>
3. Biswas P, Brummell DA. Chilling injury. In: *Postharvest physiological disorders in fruits and vegetables*, Eds. De Freita, S.T. and Pareek, S. CRC Press, Boca Raton, Florida, US. 2019:61-88.
4. Biswas P, East AR, Hewett EW. et al. Intermittent warming in alleviating chilling injury—a potential technique with commercial constraint. *Food Bioprocess Technol.* 2016;**9**:1-5. <10.1007/s11947-015-1588-7>
5. Luengwilai K, Beckles DM, Saltveit ME. Chilling-injury of harvested tomato (*Solanum lycopersicum* L.) cv. Micro-Tom fruit is reduced by temperature pre-treatments. *Postharvest Biol. Technol.* 2012;**63**:123-8. <10.1016/j.postharvbio.2011.06.017>
6. Lurie S. Genomic and transcriptomic studies on chilling injury in peach and nectarine. *Postharvest Biol. Technol.* 2021;**174**:111444. <10.1016/j.postharvbio.2020.111444>
7. Leisso RS, Gapper NE, Mattheis JP. et al. Gene expression and metabolism preceding soft scald, a chilling injury of ‘Honeycrisp’ apple fruit. *BMC Genom.* 2016;**17**:1-23. <10.1186/s12864-016-3019-1>
8. Raimbault AK, Marie-Alphonsine PA, Horry JP. et al. Polyphenol oxidase and peroxidase expression in four pineapple varieties (*Ananas comosus* L.) after a chilling injury. *J. Agric. Food Chem.* 2011;**59**:342-8. <10.1021/jf102511z>
9. Liu J, Li F, Li T. et al. Fibroin treatment inhibits chilling injury of banana fruit via energy regulation. *Sci. Hortic.* 2019;**248**:8-13. <10.1016/j.scienta.2018.12.052>
10. Jiao J, Jin M, Liu H. et al. Application of melatonin in kiwifruit (*Actinidia chinensis*) alleviated chilling injury during cold storage. *Sci. Hortic.* 2022;**296**:110876. <10.1016/j.scienta.2022.110876>
11. Huang Q, Qian X, Jiang T. et al. Effect of eugenol fumigation treatment on chilling injury and *CBF* gene expression in eggplant fruit during cold storage. *Food Chem.* 2019;**292**:143-50. <10.1016/j.foodchem.2019.04.048>
12. Cen H, Lu R, Zhu Q, et al. Nondestructive detection of chilling injury in cucumber fruit using hyperspectral imaging with feature selection and supervised classification. *Postharvest Biol. Technol.* 2016;**111**:352-61. <10.1016/j.postharvbio.2015.09.027>
13. Albornoz K, Zhou J, Yu J. et al. Dissecting postharvest chilling injury through biotechnology. *Curr. Opin. Biotechnol.* 2022;**78**:102790. <10.1016/j.copbio.2022.102790>
14. FAO. 2011. Global Food Losses and Food Waste – Extent, Causes and Prevention. <http://www.fao.org/docrep/014/mb060e/mb060e00.pdf>
15. Shipman EN, Yu J, Zhou J. et al. Can gene editing reduce postharvest waste and loss of fruit, vegetables, and ornamentals?. *Hortic. Res.* 2021;**8**. <10.1038/s41438-020-00428-4>
16. Diehl DC, Sloan NL, Bruhn CM. et al. Exploring produce industry attitudes: Relationships between postharvest handling, fruit flavor, and consumer purchasing. *Horttechnology*. 2013;**23**:642-50. <10.21273/HORTTECH.23.5.642>
17. Fernqvist F, Hunter E. Who's to blame for tasteless tomatoes? The effect of tomato chilling on consumers' taste perceptions. *Eur. J. Hortic. Sci.* 2012;**77**:193.
18. Parkin KL, Marangoni A, Jackman RL. et al. Chilling injury. A review of possible mechanisms. *J. Food Biochem.* 1989;**13**:127-53. <10.1111/j.1745-4514.1989.tb00389.x>
19. Lukatkin AS, Brazaityte A, Bobinas C. Chilling injury in chilling-sensitive plants: a review. *Agric.* 2012;**99**:111-24. <http://zemdirbyste-agriculture.lt/99(2)tomas/99_2_tomas_str1.pdf>
20. Lyons JM. Chilling injury in plants. *Ann. Rev. Plant Physiol.* 1973;**24**:445-66. <10.1146/annurev.pp.24.060173.002305>
21. Zhang W, Jiang H, Cao J. et al. Advances in biochemical mechanisms and control technologies to treat chilling injury in postharvest fruits and vegetables. *Trends Food Sci. Technol.* 2021;**113**:355-65. <10.1016/j.tifs.2021.05.009>
22. Aghdam MS, Bodbodak S. Physiological and biochemical mechanisms regulating chilling tolerance in fruits and vegetables under postharvest salicylates and jasmonates treatments. *Sci. Hortic.* 2013;**156**:73-85. <10.1016/j.scienta.2013.03.028>
23. Zhang W, Cao J, Fan X. et al. Applications of nitric oxide and melatonin in improving postharvest fruit quality and the separate and crosstalk biochemical mechanisms. *Trends Food Sci. Technol.* 2020;**99**:531-41. <10.1016/j.tifs.2020.03.024>
24. Aghdam MS, Sevillano L, Flores FB. et al. Heat shock proteins as biochemical markers for postharvest chilling stress in fruits and vegetables. *Sci. Hortic.* 2013;**160**:54-64. <10.1016/j.scienta.2013.05.020>
25. Shan Y, Zhang D, Luo Z, et al. Advances in chilling injury of postharvest fruit and vegetable: Extracellular ATP aspects. *Compr. Rev. Food Sci. Food Saf.* 2022;**21**:4251-73. <10.1111/1541-4337.13003>
26. Valenzuela JL, Manzano S, Palma F. et al. Oxidative stress associated with chilling injury in immature fruit: postharvest technological and biotechnological solutions. *Int. J. Mol. Sci.* 2017;**18**:1467. <10.3390/ijms18071467>
27. Matthus E, Ning Y, Shafiq F. et al. Phosphate-deprivation and damage signalling by extracellular ATP. *Front. Plant Sci.* 2023;**13**:1098146. <10.3389/fpls.2022.1098146>
28. Shan Y, Zhang S, Li Y. et al. The roles of exogenous ATP in postharvest fruit and vegetable: A systematic meta-analysis. *Postharvest Biol. Technol.* 2023;**199**:112305. <10.1016/j.postharvbio.2023.112305>
29. Sun J, Zhang CL, Deng SR. et al. An ATP signalling pathway in plant cells: extracellular ATP triggers programmed cell death in *Populus euphratica*. *Plant Cell Environ.* 2012;**35**:893-916. <10.1111/j.1365-3040.2011.02461.x>
30. Zhang L, Wang JW, Zhou X. et al. Effect of ATP treatment on enzymes involved in energy and lipid metabolisms accompany peel browning of ‘Nanguo’ pears during shelf life after low temperature storage. *Sci. Hortic.* 2018;**240**:446-52. <10.1016/j.scienta.2018.06.036>
31. Kim SY, Sivaguru M, Stacey G. Extracellular ATP in plants. Visualization, localization, and analysis of physiological significance in growth and signaling. *Plant Physiol.* 2006;**142**:984-92. <10.1104/pp.106.085670>
32. Wang J, Zhou X, Zhou Q. et al. Low temperature conditioning alleviates peel browning by modulating energy and lipid metabolisms of ‘Nanguo’ pears during shelf life after cold storage. *Postharvest Biol. Technol.* 2017;**131**:10-5. <10.1016/j.postharvbio.2017.05.001>
33. Middleton DA, Hughes E, Esmann M. The conformation of ATP within the Na,K‐ATPase nucleotide site: A statistically constrained analysis of REDOR solid‐state NMR data. *Angew. Chem.* 2011;**123**:7179-82. <10.1002/ange.201100736>
34. Feng JH, Wei KZ, Gao JP. et al. Determination of adenosine phosphates in mouse myocardium tissue by HPLC with UV detection and using porous graphite carbon column. *J. Chromatogr. B*. 2020;**1145**:122110. <10.1016/j.jchromb.2020.122110>
35. Ihssen J, Jovanovic N, Sirec T. et al. Real-time monitoring of extracellular ATP in bacterial cultures using thermostable luciferase. *PLoS One*, 2021;**16**:e0244200. <10.1371/journal.pone.0244200>
36. Zhou J, Fan C, Liu K. et al. Extracellular ATP is involved in the initiation of pollen germination and tube growth in *Picea* *meyeri*. *Trees*. 2015;**29**:563-74. <10.1007/s00468-014-1135-6>
37. He Y, Xiong LH, Xing XJ. et al. An ultra-high sensitive platform for fluorescence detection of micrococcal nuclease based on grapheneoxide. *Biosens. Bioelectron.* 2013;**42**:467-73. <10.1016/j.bios.2012.10.045>
38. Ji D, Wang H, Ge J. et al. Label-free and rapid detection of ATP based on structure switching of aptamers. *Anal. Biochem.* 2017;**526**:22-8. <10.1016/j.ab.2017.03.011>
39. Sang F, Zhang X, Liu J. et al. A label-free hairpin aptamer probe for colorimetric detection of adenosine triphosphate based on the anti-aggregation of gold nanoparticles. *Spectrochim. Acta A Mol. Biomol. Spectrosc.* 2019;**217**:122-7. <10.1016/j.saa.2019.03.081>
40. Liu X, Zhao Y, Ding Y. et al. Stabilization of gold nanoparticles by hairpin DNA and implications for label-free colorimetric biosensors. *Langmuir*. 2022;**38**:5542-9. <10.1021/acs.langmuir.2c00119>
41. Duong NTC, Uthairatanakij A, Laohakunjit N. et al. An innovative single step of cross-linked alginate-based edible coating for maintaining postharvest quality and reducing chilling injury in rose apple cv.'Tabtimchan' (*Syzygium* *samarangenese*). *Sci. Hortic.* 2022;**292**:110648. <10.1016/j.scienta.2021.110648>
42. Li T, Yun Z, Wu Q. et al. Proteomic profiling of 24-epibrassinolide-induced chilling tolerance in harvested banana fruit. *J. Proteom.* 2018;**187**:1-2. <10.1016/j.jprot.2018.05.011>
43. Ali ZM, Chin LH, Marimuthu M. et al. Low temperature storage and modified atmosphere packaging of carambola fruit and their effects on ripening related texture changes, wall modification and chilling injury symptoms. *Postharvest Biol. Technol.* 2004;**33**:181-92. <10.1016/j.postharvbio.2004.02.007>
44. Wang B, Zhu S. Pre-storage cold acclimation maintained quality of cold-stored cucumber through differentially and orderly activating ROS scavengers. *Postharvest Biol. Technol.* 2017;**129**:1-8. <10.1016/j.postharvbio.2017.03.001>
45. Alba-Jiménez JE, Benito-Bautista P, Nava GM. et al. Chilling injury is associated with changes in microsomal membrane lipids in guava fruit (*Psidium guajava* L.) and the use of controlled atmospheres reduce these effects. *Sci. Hortic.* 2018;**240**:94-101. <10.1016/j.scienta.2018.05.026>
46. Gwanpua SG, Jabbar A, Zhao M. et al. Investigating the potential of dual temperature storage as a postharvest management practice to mitigate chilling injury in kiwifruit. *Int. J. Refrig.* 2018;**86**:62-72. <10.1016/j.ijrefrig.2017.12.004>
47. Liao L, Li S, Li Y. et al. Pre-or post-harvest treatment with meja improves post-harvest storage of lemon fruit by stimulating the antioxidant system and alleviating chilling injury. *Plants*. 2022;**11**:2840. <10.3390/plants11212840>
48. Cao S, Yang Z, Cai Y. et al. Fatty acid composition and antioxidant system in relation to susceptibility of loquat fruit to chilling injury. *Food Chem.* 2011;**127**:1777-83. <10.1016/j.foodchem.2011.02.059>
49. Wang D, Chen Q, Chen W. et al. Melatonin treatment maintains quality and delays lignification in loquat fruit during cold storage. *Sci. Hortic.* 2021:**284**:110126. [doi:](https://doi.org/10.1016/j.scienta.2021.110126)<10.1016/j.scienta.2021.110126>
50. Sivankalyani V, Sela N, Feygenberg O. et al. Transcriptome dynamics in mango fruit peel reveals mechanisms of chilling stress. *Front. Plant Sci.* 2016;**7**:1579. <10.3389/fpls.2016.01579>
51. Bhardwaj R, Aghdam MS, Arnao MB. et al. Melatonin alleviates chilling injury symptom development in mango fruit by maintaining intracellular energy and cell wall and membrane stability. *Front. Nutr.* 2022;**9**:936932. <10.3389/fnut.2022.936932>
52. Bhardwaj R, Pareek S, Saravanan C. et al. Contribution of pre-storage melatonin application to chilling tolerance of some mango fruit cultivars and relationship with polyamines metabolism and γ-aminobutyric acid shunt pathway. *Environ. Exp. Bot.* 2022;**194**:104691. [doi:](https://doi.org/10.1016/j.envexpbot.2021.104691)<10.1016/j.envexpbot.2021.104691>
53. Patel MK, Fanyuk M, Feyngenberg O. et al. Phenylalanine induces mango fruit resistance against chilling injuries during storage at suboptimal temperature. *Food Chem.* 2023;**405**:134909. [doi:](https://doi.org/10.1016/j.foodchem.2022.134909)<10.1016/j.foodchem.2022.134909>
54. Ghasemnezhad M, Marsh K, Shilton R. et al. Effect of hot water treatments on chilling injury and heat damage in ‘Satsuma’ mandarins: Antioxidant enzymes and vacuolar ATPase, and pyrophosphatase. *Postharvest Biol. Technol.* 2008;**48**:364-71. <10.1016/j.postharvbio.2007.09.014>
55. Habibi F, Guillén F, Serrano M. et al. Postharvest treatment with glycine betaine enhances chilling tolerance of blood orange fruit by increasing antioxidant defence systems and osmoregulation during cold storage. *Sci. Hortic.* 2022;**305**:111352. <10.1016/j.scienta.2022.111352>
56. Rahmanian A, Mireei SA, Sadri S. et al. Application of biospeckle laser imaging for early detection of chilling and freezing disorders in orange. *Postharvest Biol. Technol.* 2020;**162**:111118. <10.1016/j.postharvbio.2020.111118>
57. Rodrigues C, Gaspar PD, Simões MP. et al. Review on techniques and treatments toward the mitigation of the chilling injury of peaches. *J. Food Process. Preserv.* 2022;**46**:e14358. <10.1111/jfpp.14358>
58. Sati H, Bhardwaj R, Fawole OA. et al. Postharvest melatonin application preserves quality and imparts chilling tolerance in peaches. *J. Food Biochem.* **2023**. <10.1155/2023/8126640>
59. Zhao H, Jiao W, Cui K. et al. Near-freezing temperature storage enhances chilling tolerance in nectarine fruit through its regulation of soluble sugars and energy metabolism. *Food Chem.* 2019;**289**:426-35. <10.1016/j.foodchem.2019.03.088>
60. Deng Z, Jung J, Simonsen J. et al. Cellulose nanocrystals pickering emulsion incorporated chitosan coatings for improving storability of postharvest Bartlett pears (*Pyrus communis*) during long-term cold storage. *Food Hydrocoll.* 2018;**84**:229-37. <10.1016/j.foodhyd.2018.06.012>
61. Wei C, Ma L, Cheng Y., et al. Exogenous ethylene alleviates chilling injury of ‘Huangguan’ pear by enhancing the proline content and antioxidant activity. *Sci. Hortic.* 2019;**257**:108671. <10.1016/j.scienta.2019.108671>
62. Shadmani N, Ahmad SH, Saari N. et al. Chilling injury incidence and antioxidant enzyme activities of *Carica* *papaya* L. ‘Frangi’as influenced by postharvest hot water treatment and storage temperature. *Postharvest Biol. Technol.* 2015;**99**:114-9. <10.1016/j.postharvbio.2014.08.004>
63. Zhang M, Zhang Q, Tian C. et al. Physiological and transcriptome analyses of CaCl_2_ treatment to alleviate chilling injury in pineapple. *Plants*. 2022;**11**:2215. <10.3390/plants11172215>
64. Xu R, Wang L, Li K. et al. Integrative transcriptomic and metabolomic alterations unravel the effect of melatonin on mitigating postharvest chilling injury upon plum (cv. Friar) fruit. *Postharvest Biol. Technol.* 2022;**186**:111819. <10.1016/j.postharvbio.2021.111819>
65. Ehteshami S, Abdollahi F, Ramezanian A. et al. Enhanced chilling tolerance of pomegranate fruit by edible coatings combined with malic and oxalic acid treatments. *Sci. Hortic.* 2019;**250**:388-98. <10.1016/j.scienta.2019.02.075>
66. Mishra V, Kaplan Y, Ginzberg I. Mitigating chilling injury of pomegranate fruit skin. *Sci. Hortic.* 2022;**304**:111329. <10.1016/j.scienta.2022.111329>
67. Mirshekari A, Madani B, Yahia EM. et al. Postharvest melatonin treatment reduces chilling injury in sapota fruit. *J. Sci. Food Agric.* 2020;**100**:1897-903. <10.1002/jsfa.10198>
68. Rai A, Kumari K, Vashistha P. Umbrella review on chilling injuries: Post-harvest issue, cause, and treatment in tomato. *Sci. Hortic.* 2022;**293**:110710. <10.1016/j.scienta.2021.110710>
69. Li S, Wang L, Hao Y. et al. An ultrasensitive colorimetric aptasensor for ATP based on peptide/Au nanocomposites and hemin–G-quadruplex DNAzyme. *RSC Adv.* 2014;**4**:23185-23190. <10.1039/C4RA02823F>
70. Huo Y, Qi L, Lv XJ. et al. A sensitive aptasensor for colorimetric detection of adenosine triphosphate based on the protective effect of ATP-aptamer complexes on unmodified gold nanoparticles. *Biosens. Bioelectron.* 2016;**78**:315-20. <10.1016/j.bios.2015.11.043>
71. Zhan Z, Cai J, Wang Q. et al. Green synthesis of fluorescence carbon nanoparticles from yum and application in sensitive and selective detection of ATP. *Luminescence*. 2016;**31**:626-32. <10.1002/bio.3002>
72. Zhu Y, Hu XC, Shi S. et al. Ultrasensitive and universal fluorescent aptasensor for the detection of biomolecules (ATP, adenosine and thrombin) based on DNA/Ag nanoclusters fluorescence light-up system. *Biosens. Bioelectron.* 2016;**79**:205-12. <10.1016/j.bios.2015.12.015>
73. Mao Y, Fan T, Gysbers R. et al. A simple and sensitive aptasensor for colorimetric detection of adenosine triphosphate based on unmodified gold nanoparticles. *Talanta*. 2017;**168**:279-85. <10.1016/j.talanta.2017.03.014>
